# Heart Rate Monitoring in Team Sports—A Conceptual Framework for Contextualizing Heart Rate Measures for Training and Recovery Prescription

**DOI:** 10.3389/fphys.2018.00639

**Published:** 2018-05-31

**Authors:** Christoph Schneider, Florian Hanakam, Thimo Wiewelhove, Alexander Döweling, Michael Kellmann, Tim Meyer, Mark Pfeiffer, Alexander Ferrauti

**Affiliations:** ^1^Faculty of Sport Science, Ruhr-University Bochum, Bochum, Germany; ^2^School of Human Movement and Nutrition Sciences, The University of Queensland, St. Lucia, QLD, Australia; ^3^Institute of Sports and Preventive Medicine, Saarland University, Saarbrücken, Germany; ^4^Institute of Sport Science, Johannes-Gutenberg University, Mainz, Germany

**Keywords:** player monitoring, cardiac autonomic nervous system, individual response, smallest worthwhile change, multivariate analysis, decision-making

## Abstract

A comprehensive monitoring of fitness, fatigue, and performance is crucial for understanding an athlete's individual responses to training to optimize the scheduling of training and recovery strategies. Resting and exercise-related heart rate measures have received growing interest in recent decades and are considered potentially useful within multivariate response monitoring, as they provide non-invasive and time-efficient insights into the status of the autonomic nervous system (ANS) and aerobic fitness. In team sports, the practical implementation of athlete monitoring systems poses a particular challenge due to the complex and multidimensional structure of game demands and player and team performance, as well as logistic reasons, such as the typically large number of players and busy training and competition schedules. In this regard, exercise-related heart rate measures are likely the most applicable markers, as they can be routinely assessed during warm-ups using short (3–5 min) submaximal exercise protocols for an entire squad with common chest strap-based team monitoring devices. However, a comprehensive and meaningful monitoring of the training process requires the accurate separation of various types of responses, such as strain, recovery, and adaptation, which may all affect heart rate measures. Therefore, additional information on the training context (such as the training phase, training load, and intensity distribution) combined with multivariate analysis, which includes markers of (perceived) wellness and fatigue, should be considered when interpreting changes in heart rate indices. The aim of this article is to outline current limitations of heart rate monitoring, discuss methodological considerations of univariate and multivariate approaches, illustrate the influence of different analytical concepts on assessing meaningful changes in heart rate responses, and provide case examples for contextualizing heart rate measures using simple heuristics. To overcome current knowledge deficits and methodological inconsistencies, future investigations should systematically evaluate the validity and usefulness of the various approaches available to guide and improve the implementation of decision-support systems in (team) sports practice.

## Introduction

Successful training and recovery management aims at optimizing adaptation and overall preparedness for enhanced competitive performance (Buchheit, 2014; Cardinale and Varley, 2017; Coutts et al., 2018; Kellmann et al., 2018). Monitoring the training dose and athletes' responses (e.g., fitness, fatigue, performance, and wellness) is crucial in making informed decisions on training and recovery prescriptions (Halson, 2014; Bourdon et al., 2017; McGuigan, 2017; Coutts et al., 2018; Kellmann et al., 2018). Current technological developments in the field of wearable sensors enable steady improvement in the quantification of internal- and external-load indicators during athletic activity and expand the variety of tools available to measure training responses (Cardinale and Varley, 2017). Ideally, a comprehensive monitoring system includes markers for all relevant physiological and psychological aspects of training and performance, combining them into a holistic approach (Heidari et al., 2018). Nevertheless, the handling of collected data poses a great challenge for researchers and practitioners, and available analytical strategies have rarely been systematically investigated (Thorpe et al., 2017). In this context, it is necessary to clarify how the individual longitudinal data can be analyzed on the one hand, and in which form the various parameters should be linked to one another, on the other hand.

Because team sport performance is a complex and multidimensional construct, comprehensive monitoring is crucial in understanding athletes' training response to modify training and recovery strategies (Halson, 2014; Bourdon et al., 2017; McGuigan, 2017; Coutts et al., 2018). Moreover, team sport coaches and practitioners usually deal with a large number of athletes. Another great challenge is, therefore, the implementation of a simple but effective monitoring system that involves at least some measures of training load, wellness, fitness, and readiness (Gabbett et al., 2017; McGuigan, 2017). The frequent assessment of various metrics could be difficult as compliance can be affected by the busy schedule and complex requirements of the team sport athlete.

In this regard, the use of heart rate (HR) and heart rate variability (HRV) measures in sports have been discussed for decades, as they represent an inexpensive, time-efficient, and non-invasive method to monitor the status of the autonomic nervous system (ANS) and cardiovascular fitness (Achten and Jeukendrup, 2003; Aubert et al., 2003; Borresen and Lambert, 2008; Alexandre et al., 2012; Daanen et al., 2012; Buchheit, 2014). Despite the large body of research and possible applications, monitoring athletes' training responses with HR measures is not widely implemented (Buchheit, 2014), which is due in part to contradictory findings (Alexandre et al., 2012; Bellenger et al., 2016), methodological inconsistencies (Plews et al., 2013), or partial misinterpretations (e.g., assuming that HR measures can reflect overall fatigue or fitness directly) (Achten and Jeukendrup, 2003; Buchheit, 2014). In any case, it is indisputable that HR data can measure only a limited number of aspects of performance or training response, and therefore must be combined with additional parameters.

In this technology report, we first briefly outline current applications and limitations of monitoring training response with HR and HRV in team sport athletes. Second, we present a conceptual framework for contextualizing HR measures, and methodological considerations of univariate and multivariate analysis approaches of HR monitoring data are addressed. Finally, we illustrate how different analysis concepts may affect the evaluation of data, and provide two case examples for practical decision-making with a simple, multivariate heuristical approach.

## HR monitoring in athletes

HR measures are used as surrogate markers of the *cardiac* ANS status (Aubert et al., 2003; Michael et al., 2017). As the ANS is interlinked with many physiological systems, HR measures might reflect (aerobic-based) adaptation and fatigue status (Buchheit, 2014; Hottenrott and Hoos, 2017; Thorpe et al., 2017). However, HR measures are determined by multiple influencing factors, such as environmental (e.g., noise, light, temperature), physiological (e.g., cardiac morphology, plasma volume, autonomic activity), pathological (e.g., cardiovascular disease), psychological (e.g., mood, emotions, stress) conditions, and non-modifiable factors (e.g., age, sex, ethnicity), as well as lifestyle (e.g., fitness, sleep, medication, tobacco, alcohol) and determinants of physical activity (e.g., intensity, duration, modality, economy, body position) (Sandercock et al., 2005; Buchheit, 2014; Fatisson et al., 2016; Sessa et al., 2018). Nevertheless, it is assumed that, in competitive sports, the influence of training plays a predominant role in ANS status changes and, therefore, HR measures might be able to represent the athlete's training status (Lamberts et al., 2010; Buchheit, 2014).

The large number of original and review articles on HR monitoring published in recent decades documents the high interest in exercise and sport science (Task Force, 1996; Achten and Jeukendrup, 2003; Aubert et al., 2003; Carter et al., 2003; Sandercock et al., 2005; Hottenrott et al., 2006; Borresen and Lambert, 2008; Bosquet et al., 2008; Alexandre et al., 2012; Daanen et al., 2012; Plews et al., 2013; Stanley et al., 2013; Buchheit, 2014; Hettinga et al., 2014; Bellenger et al., 2016; Kingsley and Figueroa, 2016; Berkelmans et al., 2017). The growing popularity of HR measures among practitioners (Akenhead and Nassis, 2016; Thorpe et al., 2017), combined with the increasing number of commercial products and software for HR recording and analysis (Naranjo et al., 2015; Flatt and Esco, 2016; Perrotta et al., 2017; Plews et al., 2017b) further highlights the practical significance of this research field. While relying on countless years of scientific and practical experience (Israel, 1982), no other physiological parameters are available that provide a non-invasive, time-efficient, cost-effective, and continuous insight into a human's physiological response in almost any environment or stress situation. Nevertheless, HR measures cannot address all aspects of performance, fatigue, and well-being, but are mainly reflective of ANS status and cardiovascular fitness (Buchheit, 2014).

### HR measures and protocols

Heart activity (HR and stroke volume) is integrated into numerous feedback (e.g., muscle mechanoreceptors) and feedforward (e.g., “central command”) loops, and is continuously modulated by ANS activity on a beat-to-beat basis (Michael et al., 2017). Thus, it is critical to consider standardized procedures when collecting, analyzing, and comparing HR and HRV [HR(V)] within or between athletes. All HR measures are somehow related to ANS activity, but differ in their physiological determinants and their time course of adaptation, and display different sensitivity to changes in fitness, performance and training load (Bosquet et al., 2008; Buchheit, 2014). In this chapter (HR Monitoring in Athletes), we refrain from a detailed survey of the literature, as many review articles have already described the relationships between HR measures, the ANS, and other influencing factors, and have further defined general methodological guidelines for data collection and preparation. For example, an excellent overview of monitoring training status with HR measures has been provided by Buchheit (2014). Nevertheless, we provide a brief and focused account of the application and limitations of HR monitoring in team sports.

#### Resting measures

Supine or seated short-term (5–10 min, Task Force, 1996) resting HR measures (HRrest, HRVrest) are currently suggested as a best practice for monitoring an athlete's ANS status (Buchheit, 2014). Resting HR(V) can be directly influenced by short-term (e.g., blood/plasma volume changes, fatigue) and long-term training responses (e.g., cardiac morphology), which in turn may obscure the observation of changes in ANS activity (Fellmann, 1992; Zavorsky, 2000; Achten and Jeukendrup, 2003; Buchheit, 2014). Resting measurements (during nocturnal sleep or after awakening) are attractive since they are characterized by a high degree of standardization and, therefore, minimize many confounding factors (e.g., previous activity, time of day) (Achten and Jeukendrup, 2003; Fatisson et al., 2016). Additionally, these measurements can also be collected on resting days, in case of injury or sickness, and can further be used to modify individual training and recovery plans before the first daily session (Buchheit, 2014). Although some authors suggest that resting HRV might be more sensitive to training status than resting HR (Naranjo et al., 2015; Flatt and Esco, 2016), the superiority of HRVrest could be neither confirmed nor rejected (Billman et al., 2015). There are still large methodological inconsistencies in HRV assessment that impede the comparison and summary of findings (Task Force, 1996; Bellenger et al., 2016).

In team sports, daily morning assessments may prove useful, especially in short- to mid-term periods of increased stress, such as the evaluation of pronounced travel loads or training camps (Fowler et al., 2017; Malone et al., 2017). Under field conditions, time-domain HRV indices (e.g., Ln rMSSD: natural logarithm of the square root of the mean squared differences of successive normal R-R intervals) have become established to assess daily changes in ANS status, as they are more reliable (Al Haddad et al., 2011) and less affected by different breathing patterns (Penttilä et al., 2001; Saboul et al., 2013) compared to spectral analyses. When assessing long-term changes, it is suggested to analyze (rolling) weekly averages (≥3–4 measurements per week) to increase validity (Plews et al., 2014) and express day-to-day-fluctuations as a weekly coefficient of variation (CV; Plews et al., 2012; Flatt and Esco, 2016). However, it might be unrealistic in practice to implement frequent (≥3–4 times per week) home-based resting measures in an entire squad of elite or high-level players over a prolonged training period (Buchheit, 2014; Thorpe et al., 2017). An alternative approach could use pre-training recordings (Nakamura et al., 2016; Malone et al., 2017). Furthermore, the extended evaluation and application of ultra-short-term recordings (<5 min, often ≤1 min; Flatt and Esco, 2013; Esco and Flatt, 2014; Nakamura et al., 2015; Pereira et al., 2016; Esco et al., 2018) with commercial software, such as smartphone applications (e.g., *Elite HRV* Perrotta et al., 2017; *ithlete* Flatt and Esco, 2013; *HRV4Training* Plews et al., 2017b), enables feasible analysis of an entire team's data almost immediately after the assessment. These technological developments may improve compliance and increase the applicability of resting measurements in the future, at least in settings with high formal program commitment as in junior or high school and college athletes.

#### Exercise measures

Over a wide range of endurance exercise intensities, exercise HR (HRex) is linearly related to oxygen uptake and energy expenditure during continuous work and is therefore commonly used to monitor and prescribe exercise intensity and training load (Achten and Jeukendrup, 2003; Borresen and Lambert, 2009; Alexandre et al., 2012; Berkelmans et al., 2017). Furthermore, exercise HR has been traditionally evaluated under submaximal (HRex) and maximal efforts (HRmax) using incremental tests to assess cardiovascular fitness (Achten and Jeukendrup, 2003; Buchheit, 2014). As the relationship between common (vagal-related) HRV measures and exercise intensity is flawed (Buchheit, 2014; Michael et al., 2017; see also section Limitations of Univariate HR Monitoring) and beat-to-beat recordings during exercise are susceptible to artifacts (e.g., lost beats due to HR belt movement), only HRex at fixed external loads (not exercise HRV) averaged over the last 30-60 s can be recommended for longitudinal athlete monitoring (Buchheit, 2014). Whether exercise HR can depict fitness impairments sensitively is still unclear, as increased HRex does not indicate impaired performance *per se* (Buchheit, 2014; Thorpe et al., 2017) but likely occurs with prolonged detraining (Mujika and Padilla, 2000a,b). Moreover, similar to interpreting changes in resting HR(V), long-term fitness-related changes in HRex may also be skewed due to acute or short-term responses to training or environmental conditions.

Since the repeated assessment of maximal physical performance is unsuitable in (team sport) athletes, submaximal, non-exhaustive tests have been more frequently adopted by researchers and practitioners during recent decades (Buchheit, 2014; Halson, 2014; Akenhead and Nassis, 2016; Capostagno et al., 2016; Thorpe et al., 2017). However, the protocols used vary greatly in modality (running Malone et al., 2017 vs. cycling Thorpe et al., 2015), load characteristics (continuous Buchheit et al., 2010 vs. intermittent Brink et al., 2013, linear Buchheit et al., 2010 vs. shuttle runs Bradley et al., 2011, constant Buchheit et al., 2010 vs. graded Bradley et al., 2011), test duration (5 min Buchheit et al., 2010 to 16 min Vesterinen et al., 2017), intensity (low-intensity Buchheit et al., 2013c vs. high-intensity Vesterinen et al., 2017) and workload prescription (standardized Bradley et al., 2011 vs. individualized Buchheit et al., 2010, internal Vesterinen et al., 2017 vs. external Bradley et al., 2011).

**Figure 1:**
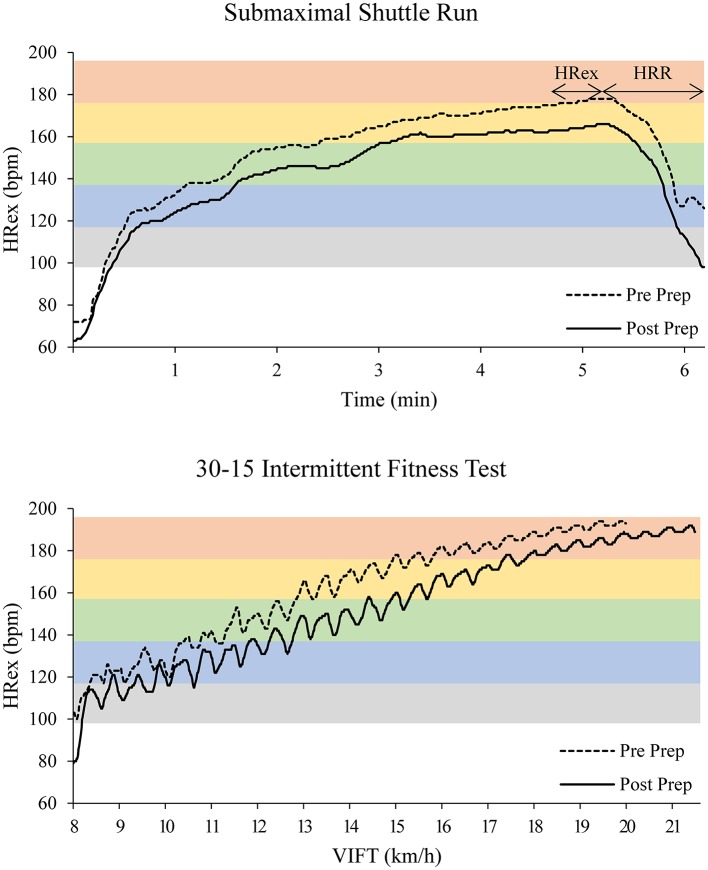
Example of heart rate (HR) recordings during submaximal and maximal shuttle runs as part of preseason performance testing in a semi-professional basketball player. Performance testing was conducted at the beginning and the end of an 8-week preseason preparation period for a 25-year-old semi-professional basketball player. The submaximal shuttle run consisted of 5 min of running (~1, 1, and 3 min at 9.0, 10.5, and 12.0 km/h, respectively; 28 m shuttle length) followed by 1 min of passive recovery and was performed as the first part of the warm-up. Maximum (aerobic) fitness was assessed using an incremental field test (30-15 IFT, 30-15 Intermittent Fitness Test, Buchheit, 2008) at the end of each session. The player showed a 1.5 km/h increase in maximum running speed (VIFT), a 13 bpm decrease in exercise HR during, and a 16 bpm increase in HRR following, the submaximal shuttle run. The colored horizontal bars represent 10%-wide HR zones starting at 50%HRmax (e.g., red bar: 90–100%HRmax). HRex: exercise HR; HRR: HR recovery over 60 s; Prep: preparation period.

In team sports, standardized (rather than individualized) submaximal running tests seem to be most appropriate in a variety of settings (level of competition, team budget, squad size). Low-intensity exercise could be implemented in the first part of the warm-up for most athletes (fit, unfit, fatigued, early stage of return to activity after an injury or sickness) and scenarios (training camps, preparation and recovery periods, in-season) without adding substantial fatigue, whereas higher intensities might be associated more closely with sport-specific performance (Bangsbo et al., 2008; Lamberts et al., 2010, 2011; Bradley et al., 2011). In absence of definite protocol recommendations in terms of test quality criteria (validity, reliability, signal-to-noise ratio), we suggest using either submaximal versions of established field-tests (*Multi-stage Fitness Test* Léger and Lambert, 1982, *Yo-Yo Tests* Bangsbo and Mohr, 2012, *30-15 Intermittent Fitness Test* Buchheit, 2010) or fixed-intensity runs on a specific shuttle length (or field size). Figure 1 shows exemplary HR recordings of a semi-professional basketball player during submaximal and maximal shuttle runs, which display typical changes in HRex in response to a preparation period (see figure legend for details).

#### Post-exercise measures

Following exercise cessation, HR decreases exponentially, and HRV indices start to increase. Post-exercise HR measures (HRR: HR recovery, HRVpost) reflect general hemodynamic adjustments and might be related to aerobic fitness, wellness, and readiness to perform (Buchheit, 2014). ANS activity following exercise cessation is influenced primarily by parasympathetic reactivation in the early stage of recovery [during the first minute(s)], followed by additional sympathetic withdrawal during mid- to long-term recovery (minutes to hours; Borresen and Lambert, 2008; Hottenrott and Hoos, 2017; Michael et al., 2017; Peçanha et al., 2017). However, post-exercise ANS activity and HR(V) recovery are influenced by the preceding (relative) intensity (Stanley et al., 2013; Michael et al., 2017), and may, therefore, be more indicative of fitness than ANS status (Buchheit, 2014). In general, HRR is more favorable than HRVpost. It requires shorter recording periods (HRR: 30–60 s vs. HRVpost: ≥3–5 min), is accessible with any HR device, and may have a superior signal-to-noise ratio (Buchheit, 2014). The easiest way to calculate HRR is by taking the difference of HR at exercise cessation and after, for example, 1 min recovery (Peçanha et al., 2017). However, it is recommended to average HR recordings over several seconds (typically 5–15 s) to increase objectivity and reduce (measurement) error (Daanen et al., 2012; Buchheit, 2014).

From a practical point of view, team sports practitioners should evaluate the additional effort and benefit of post-exercise measures critically in their own setting. While an additional (standing or seated) 30–60 s recording seems to be reasonable, it remains unclear whether HRR after submaximal exercise adds beneficial information (to HRex), especially when workloads are fixed rather than individualized in team sports (different relative intensities between players). Additionally, post-exercise measures could unnecessarily complicate data collection and interpretation in the worst-case scenario (see Buchheit, 2014 for discussion).

### Monitoring training response with HR measures

#### Acute responses

Monitoring an athlete's acute changes in HR measures in response to training is a critical but, at the same time, debated topic in HR(V) research. A major component of the scientific discussion is centered around day-to-day fluctuations in (especially resting) HR measures and possible causes of these variations (Buchheit, 2014). The underlying mechanisms are not entirely clear yet. There are arguments for daily changes as reflective of measurement *noise* (i.e., measurement error), which results in poor reliability of daily resting measures (Al Haddad et al., 2011) compared to exercise HR (Buchheit, 2014) and should, therefore, be interpreted as *random error*. Furthermore, day-to-day fluctuations might be interpreted as (physiological) *signal*, and changes being related to training load, stress, and fatigue (Stanley et al., 2013). In line with the latter assumption, several attempts have been made to guide training programs based on daily (resting) HRV as a marker of (cardiovascular) recovery, resulting in either larger adaptations or more efficient training compared to conventional predefined training programs (Kiviniemi et al., 2007, 2010; Vesterinen et al., 2016; da Silva et al., 2017; Nuuttila et al., 2017). However, it must be considered that HRV-guided training programs have always been exclusively based on endurance training and were subject to certain restrictions and training principles (for example, a maximum of two successive high-intensity training days).

In general, training intensity is a key determinant of *cardiac* autonomic activity alterations following aerobic-oriented exercise (e.g., the higher the intensity, the longer the homeostatic distraction) and might be more influential than duration (Stanley et al., 2013; Hottenrott and Hoos, 2017; Michael et al., 2017). Complete cardiac autonomic recovery requires up to 24 h following low-intensity, 24–48 h following threshold-intensity and at least 48 h following high-intensity endurance exercise (Stanley et al., 2013). Therefore, acute changes in training load can result in altered vagal-related HRV (Stanley et al., 2013; Malone et al., 2017; Michael et al., 2017), HRR (Borresen and Lambert, 2007; Daanen et al., 2012; Malone et al., 2017) and HRex (Buchheit et al., 2013a,c; Malone et al., 2017). Furthermore, stable (Plews et al., 2012) or reduced (Flatt and Esco, 2016) day-to-day variations (expressed as a weekly CV) in resting HRV have been observed together with positive adaptation, but also a large reduction in CV was reported before non-functional overreaching (Plews et al., 2012). However, as previously described, numerous circumstances are known to acutely affect HR indices, such as plasma volume changes [e.g., due to heat acclimatization, (intense) aerobic exercise (Fellmann, 1992)], hydration status (Achten and Jeukendrup, 2003; Buchheit, 2014), sickness (Buchheit et al., 2013c), or long-haul travel (Fowler et al., 2017), which must be considered when interpreting day-to-day changes. Typically, these acute effects are reversed within a few days.

#### Short-term responses

During short- to mid-term periods of increased stress or intensified training, such as long-haul flight travel (Fowler et al., 2017) and heat, altitude, or training camps with increased volume and/or intensity (Achten and Jeukendrup, 2003; Buchheit et al., 2011; Berkelmans et al., 2017), HR monitoring might enable practitioners to assess an athlete's ability to cope with, and recover from, the induced demands. In the context of training, all of the previously described HR measures have been shown to reflect overload-induced performance changes sensitively on several occasions (Pichot et al., 2000; Borresen and Lambert, 2007; Bosquet et al., 2008; Bellenger et al., 2016; Capostagno et al., 2016; Hammes et al., 2016; Flatt et al., 2017) and therefore are possibly reflective of short-term (i.e., cumulative) fatigue responses. For example, in unpublished studies, we observed substantially increased HRrest (decreased HRVrest) in supine position within 6-day overload microcycles of either high-intensity interval training or intensive whole-body strength training. While these changes in the supine recording position might be somewhat plausible due to the excessive overload, the standing HR(V) recordings displayed a large progressive reduction in HRrest (increased HRVrest) during the high-intensity interval training period. In the subsequent 4-day recovery phase, these alterations showed reverse trends. In summary, the changes in (supine) resting HR measures were parallel to the (stress- and fatigue-related) changes in training-specific performance (repeated sprint ability and maximal strength, respectively; see Table 1 in section Training Context is Key for further details).

#### Long-term responses

Since an athlete's training status is influenced by acute, short-term, and long-term responses, it is of central importance to consider the (aerobic) fitness level, *chronic* training loads, and the current training phase of the athlete for correct interpretation and contextualization of HR measures. In general, HR measures correlate with aerobic fitness or performance markers, with resting and exercise HR being lower and resting HRV being higher in better-trained athletes (Achten and Jeukendrup, 2003; Aubert et al., 2003; Sandercock et al., 2005; Hottenrott et al., 2006; Messina et al., 2012; Plews et al., 2013; Hottenrott and Hoos, 2017; Proietti et al., 2017; Thorpe et al., 2017; Sessa et al., 2018). However, it must be considered that increased exercise or test performance is not necessarily reflective of positive adaptation since increased “readiness” or motivation at the same fitness level may cause higher performance outcomes (Plews et al., 2013; Coutts et al., 2018). This likely contributes to some of the contraindicatory findings in research (see section Contextualizing HR Measures). Overall, fewer data exist on the sensitivity of HR measures to detect negative training response or maladaptation (Buchheit, 2014; Bellenger et al., 2016).

In trained athletes, moderate training loads typically increase aerobic fitness and HRV, whereas high training loads reduce HRV (Iellamo et al., 2002; Manzi et al., 2009; Plews et al., 2013). HRR is typically accelerated with high training volume (Buchheit, 2014). It is generally assumed that increased training volume likely results in HR(V) changes reflecting increased parasympathetic activity (e.g., decreased HRrest and increased HRVrest), whereas increased training intensity with a concomitant decrease in training volume results in HR(V) changes reflecting increased sympathetic activity (increased HRrest and decreased HRVrest) (Israel, 1982; Fry and Kraemer, 1997; Lehmann et al., 1998; Armstrong and VanHeest, 2002; Plews et al., 2013; Buchheit, 2014; Hottenrott and Hoos, 2017).

In endurance athletes, a bell-shaped time course of resting HRV in the weeks leading up to a key race may reflect an optimal scenario for peak competitive performance (Manzi et al., 2009; Plews et al., 2013, 2017a; Buchheit, 2014). Vagal-related HRV likely increases during the building phase, which is characterized by high training volume at low intensities (Buchheit, 2014). During tapering, decreased HRVrest and increased performance is typically observed, which could be explained by a shift of training distribution toward high-intensity exercise, as well as pre-competition stress (Edmonds et al., 2013; Plews et al., 2013; Buchheit, 2014). We assume that some contradictory findings on the relationship between HR measures, performance, and fatigue are caused by these observations, since neither aspects of periodization nor delayed training effects have been adequately considered in the available meta-analysis (Bosquet et al., 2008; Bellenger et al., 2016), nor has inter-individual time course of HR(V) response been properly assessed or reported, with the exception of several case studies (Plews et al., 2012, 2017a; Stanley et al., 2015). In summary, cumulative, and long-term HR(V) responses during different training phases could be explained by a prolonged accumulation of intensity-related acute effects of single training sessions in the presence or absence of sufficient recovery to reach baseline levels (Stanley et al., 2013; Buchheit, 2014). An overview of acute, short-term and long-term training responses in HR measures is provided in Table 1 (section Training Context is Key).

### Applications in team sports

In recent years, elite team sport athletes have become more exposed to high competitive loads due to the increased frequency and intensity of domestic and international competitions during both the domestic season and the off-season period (Thorpe et al., 2017). As increased player availability may lead to an increase in chances for success, fatigue management is crucial for injury and illness reduction (Bourdon et al., 2017; Thorpe et al., 2017). However, at moderate to high performance levels, there is usually a consistent and similar structure for each week during the competitive period, which may intuitively lead to weekly scheduling of training and testing relative to days until or after game-day (McGuigan, 2017; Thorpe et al., 2017). This weekly structure creates regular and comparable testing conditions (e.g., two days after competition), which may help to minimize acute “confounding” effects (e.g., fatigue) when interpreting long-term training changes in HR measures (e.g., fitness).

A large challenge in team sport monitoring is the complex and multifactorial nature of sports performance, training, and game demands, which includes technical, tactical, physiological, psychological, and social components (Coutts et al., 2018). To date, there is no uniform definition of player or team performance, which limits its quantitative description and the identification of possible influencing factors. Further, it remains speculative as to which amount the previously described associations between changes in training volume and intensity with changes in HR measures in endurance athletes are transferable to team sports, since the appropriate quantification of training load, volume, and intensity over the variety of training modalities and biological systems stressed in team sport practice is challenging (Buchheit, 2014; Bourdon et al., 2017).

**Figure 2:**
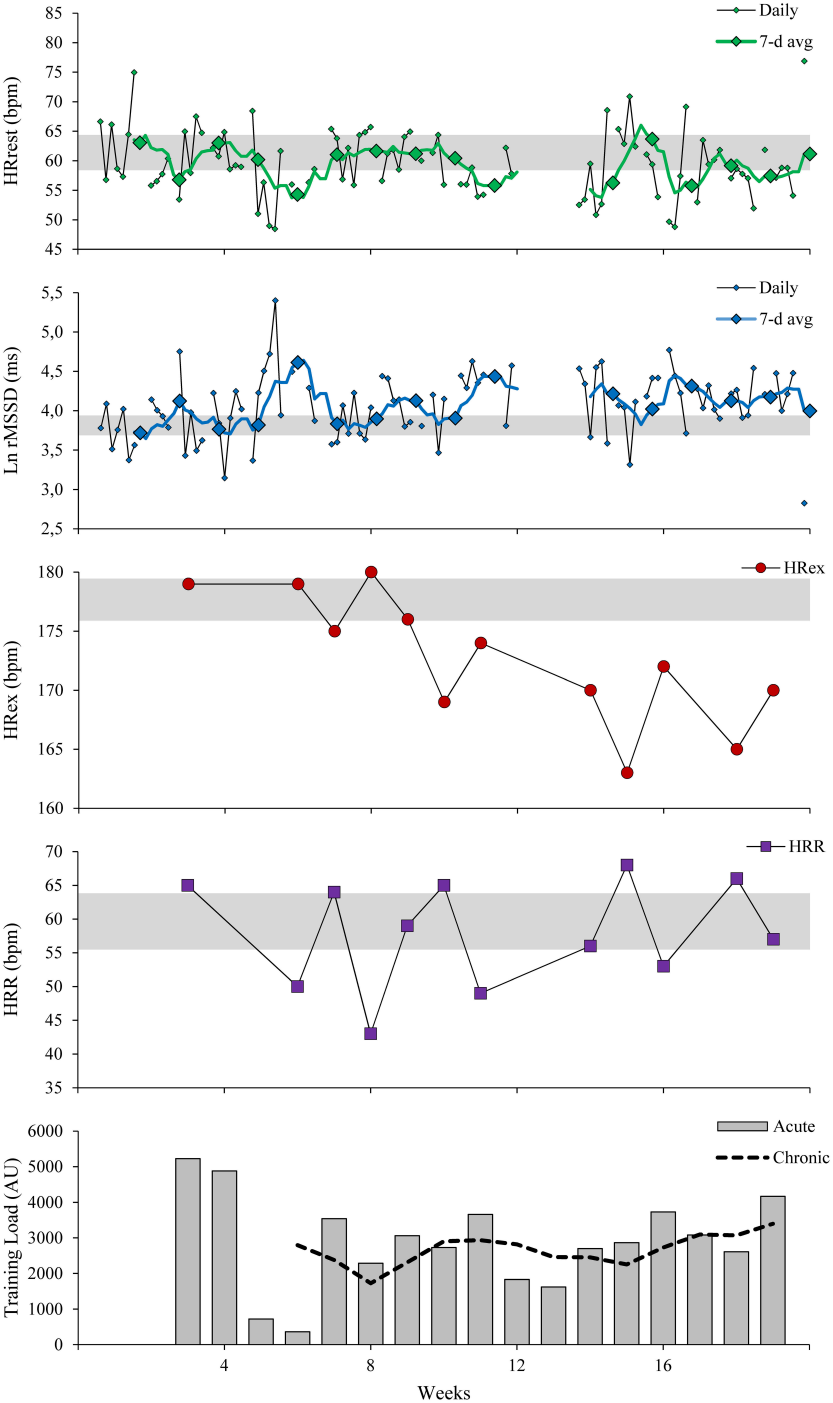
Changes in HR measures in a semi-professional basketball player during a preseason preparation period and the first half of the competitive season. Resting HR measures (HRrest, Ln rMSSD) were assessed daily with 1-min ultra-short-term recordings upon awakening, in a seated position using commercial HR monitoring software (*HRV4Training*, Plews et al., 2017b). Values are displayed as daily values and rolling 7-day averages. Exercise HR (HRex) and HR recovery (HRR) were assessed weekly with a submaximal shuttle run (see Figure 1 for details) during the warm-up in the team's evening practice 2-days post game-day. Acute and chronic training loads were calculated over 1 and 4 weeks of training, respectively [training load (AU, arbitrary units) = session-RPE (0–10) × training duration (min), (Gabbett, 2016)]. The gray horizontal bars represent trivial changes based on the suggested smallest worthwhile change for each measure: 0.5 × SD during the first 2 weeks for HRrest and HRVrest (Ln rMSSD), 1% for HRex and 7% for HRR (Buchheit, 2014).

Despite these limitations, analyzing dose-response relationships is a central component of athlete management (Gabbett et al., 2017; McLaren et al., 2018), as it helps to assess injury risk (Gabbett, 2016; Bourdon et al., 2017) and thus may indirectly influence sports performance (i.e., success) through increased player availability (Thorpe et al., 2017). Since physical performance measures during sport-specific drills and match play are highly variable, external-internal load relationships are commonly assessed using submaximal tests (Buchheit, 2014; Thorpe et al., 2017). The protocols are typically based on continuous or intermittent aerobic-based exercise (Bradley et al., 2011; Brink et al., 2013; Buchheit et al., 2013a), which are well standardized but correspondingly less valid for overall physical performance (Thorpe et al., 2017). The use of sport-specific “closed-loop” drills might be an alternative approach, as sport-specific motion patterns and demands are simulated and performance output might be less variable than during an actual match (Buchheit et al., 2013a; Malone et al., 2017; Thorpe et al., 2017). Also, developments in wearable sensor technology will enable researchers and practitioners to assess integrated external and internal loads during any sport-specific training modalities in the future (see Lacome et al., 2018 for practical example). These developments, for example, may allow (almost) real-time analysis of locomotor movement patterns on the physiological response, such as changes in running technique and, therefore, running economy on HR response. For illustrative purposes, Figure 2 represents an overview of currently suggested applications of resting and exercise HR measures in a semi-professional team sport athlete during a preparatory phase and the first half of the competitive season.

## Contextualizing HR measures

### Limitations of univariate HR monitoring

Although each of the previously described HR measures was sensitive to changes in fitness, fatigue, and performance in several instances, a recent meta-analysis found that the direction of change was the same for both increased and decreased performance (Bellenger et al., 2016). For example, vagal-related HRVrest increased parallel to both increased and decreased (aerobic) performance, representing either increased parasympathetic modulation or parasympathetic hyperactivity. Similarly, decreased HRex was observed in both concurrent performance increases (Buchheit, 2014) and overreaching-associated performance impairments (Bosquet et al., 2008). To date, the only promising approach for deciphering this dilemma lies in the contextualization of HR measures and the use of multivariate approaches (Bosquet et al., 2008; Lamberts, 2009; Plews et al., 2013; Buchheit, 2014; Bellenger et al., 2016; Capostagno et al., 2016; Bourdon et al., 2017; Hottenrott and Hoos, 2017; Thorpe et al., 2017; Coutts et al., 2018; Kellmann et al., 2018).

As previously described, a fundamental difficulty is that fatigue and performance are multifactorial constructs (Fry and Kraemer, 1997; Armstrong and VanHeest, 2002; Borresen and Lambert, 2008; Meeusen et al., 2013; Buchheit, 2014; Thorpe et al., 2017; Coutts et al., 2018; Kellmann et al., 2018), which, under certain circumstances, can be influenced measurably by changes in an athlete's ANS status (Israel, 1982; Lehmann et al., 1993) and vice versa. However, training elicits a variety of responses and adaptations on various levels (e.g., cardiovascular, hormonal, neuromuscular, psychological), any of which may result in performance or fatigue changes, either in isolation or combination. Conversely, it is unlikely that any single marker can accurately display changes in a multidimensional construct, such as performance or fatigue (Meeusen et al., 2013; Bourdon et al., 2017; Coutts et al., 2018; Kellmann et al., 2018). Therefore, HR(V) measures can only be used to assess ANS status (at rest, exercise onset, post-exercise) and overall cardiovascular function (during exercise; Buchheit, 2014) and should be considered as only one of the determinants influencing an athlete's training status.

Also, the (mathematical) relationship between ANS activity and HR(V) is indirect and is an often-overlooked limitation in research, which could cause partial misinterpretations (Plews et al., 2013; Buchheit, 2014). More precisely, this means that changes in ANS status (i.e., ANS activity) are not directly reflected in changes in HR measures, and direct associations cannot be assumed (Plews et al., 2013; Buchheit, 2014; White and Raven, 2014; Hottenrott and Hoos, 2017). For example, increasing vagal nerve activity generally increases vagal-related HRV. However, at low HR levels, HRV is often reduced rather than increased due to parasympathetic hyperactivity causing the so-called *saturation* phenomenon, which may be explained by saturation of acetylcholine receptors at the myocyte level (Plews et al., 2013; Buchheit, 2014). To overcome this issue, resting HR and HRV should be concomitantly assessed and interpreted using intraindividual historical data, representing vagal tone and modulation respectively, and normalizing HRV for the prevailing R-R interval (Plews et al., 2013; Sacha, 2013; Buchheit, 2014; Billman et al., 2015). During exercise, ANS balance continuously shifts from parasympathetic to sympathetic dominance as a function of intensity, whereas vagal-related HRV indices typically level off at moderate intensity (Buchheit, 2014; Michael et al., 2017) and therefore cannot measure ANS activity over the entire range of intensities. Furthermore, HRR and HRVpost, as possible indicators of ANS activity, might be biased by metaboreflex stimulation and should, therefore, be concomitantly interpreted with HRex (Buchheit, 2014).

### Training context is key

The most relevant information for contextualizing HR measures includes training phase, training load, and intensity distribution (Buchheit, 2014). Also, it seems necessary to consider the specific time course of training schedules and training responses and further examine (subjective) measures of well-being and recovery/fatigue state, or rating of perceived exertion (RPE) when using exercise measures. To get a more holistic impression of an athlete's training status, practitioners must combine these measures with additional markers of sport-specific performance (Bosquet et al., 2008; Lamberts, 2009; Plews et al., 2013; Buchheit, 2014; Bellenger et al., 2016; Capostagno et al., 2016; Hottenrott and Hoos, 2017; Thorpe et al., 2017). Table 1 provides an overview of changes in HR and context measures within different training settings. Particular emphasis was placed on structuring the information regarding the time course of training responses as well as the respective training context. The summarized and schematized changes reflect overall group-based effects. Typically, these observed group-effects are accompanied by large inter-individual variation, which might display contrary behavior on the individual level and highlights the necessity for individualized analysis in sports practice (Plews et al., 2013; Buchheit, 2014; Volterrani and Iellamo, 2016; Hottenrott and Hoos, 2017). However, referring to group-based suggestions of expectable changes might be an appropriate starting point if practitioners are aware of the common between-athlete variations in response and try to identify individual response patterns to consider them for future analysis.

**Table 1:** Overview and schematic representation of suggested overall effects in different HR and context measures in various (team) sports-related scenarios [data derived from reviews (R), original articles (O), monographs (M), book chapters (C) in scientific collections, and PhD theses (T)].

**Training & environmental context**	**Resting HR(V)**		**Exercise HR(V)**					**Training**	**Perception**		**Performance**	**References and comments**
	**HRrest**	**HRVrest**	**HRex**	**HRR**	**HRV post**	**HRmax**	**RPE**	**Load**	**Stress/Recovery**	**Wellness**	***(aerobic-based)***	
**ACUTE RESPONSES [WORKOUT -DAY(S)]**												
**Training sessions**												R: (Stanley et al., 2013; Kingsley and Figueroa, 2016); *assumptions based on HRV changes
Intense (endurance)	↑*	↓										
Low-intensity (endurance)	↓*	↑										
Strength	↑*	↓										
**Competition**												O: (Edmonds et al., 2013; Thorpe et al., 2016)
Game day	↑	↓										
Day(s) post game day	↑	↓	↔	↔	↔			↓	↑/↓	↓		
**Daily load changes**												R: (Mujika and Padilla, 2000a); O: (Buchheit et al., 2013a,c; Thorpe et al., 2015; Malone et al., 2017)
Increased load		↑	↓	↑(↔)	↓			↑	↑/↓	↑		
Decreased load		↓	↑	↓(↔)	↑			↓	↓/↑	↓		
**Sickness**												O: (Buchheit et al., 2013c); HR(V) recovery within 2–3 days
Day before sickness	↔	↔	↑				↔	↑		↔		
Day(s) following sickness	↑	↓	(↑)				↑			↔↓		
**Environmental conditions**												R: (Achten and Jeukendrup, 2003; Berkelmans et al., 2017); O: (Buchheit et al., 2011)
Heat, humidity, altitude/ hypoxia			↑	↔	↔		↑					
**Long-haul flight travel**												O: (Fowler et al., 2017); time difference < 8 h, >13 h respectively
Day after < 15-h flight	↓↔		↓	↓				↓	↓/	↓		
Days after >26-h flight	↑		↑	↓				↓	↓/	↓		
**SHORT-TERM RESPONSES [DAYS-WEEK(S)]**												
**Weekly load changes**												O: (Pichot et al., 2000; Borresen and Lambert, 2007; Flatt et al., 2017)
Increased weekly load	↑	↓		↓				↑				
Decreased weekly load	↓	↑		↑				↓	/↑			
**Short-term overload training (6 days, 11 sessions)**												O: (Wiewelhove et al., 2015; Hammes et al., 2016; Raeder et al., 2016); ^u^unpublished observations; *derived from power output at fixed %HRmax; () altered during first days, than reversed
High-intensity & high-volume cycling	(↑)↔su^u^	(↓)↔su^u^	↓*	↑		↓	↑	high	↑/↓	↓	↓	
	↓st^u^	↑st^u^										
High-intensity interval running	(↑)↔su^u^	(↓)↔su^u^						high	↑/↓	↓	↓RSA	
	↓st^u^	↑st^u^										
Intensive strength training	↑su^u^	↓su^u^						high	↑/↓	↓	↓Strength	
	↔st^u^	↔st^u^										
**Training in special environmental conditions**												O: (Buchheit et al., 2011, 2013a,b), T: (Flatt, 2017); *reversed/near baseline values
First days at altitude	↑	↓	↑				↑	high		↔	↓	
Altitude acclimatization	↓*	↑*	↑				↔(↑)*	high		↔(↑)*	↓(↔)	
Heat acclimatization		↑↔	↓	↔(↓)	↑	↓	↔↑	↑		↔↓	↑	
**LONG-TERM RESPONSES (WEEKS)**												
**Long-term training adaptation**												R: (Mujika and Padilla, 2000a,b; Zavorsky, 2000; Achten and Jeukendrup, 2003; Aubert et al., 2003; Carter et al., 2003; Sandercock et al., 2005; Borresen and Lambert, 2008; Daanen et al., 2012; Plews et al., 2013; Buchheit, 2014); *saturation: ↓HRV, ↓HR & ↓HRV/RR ratio
(Aerobic) endurance training	↓	↑(↓*)	↓	↑		↓↔	↓↔	↑vol & ↔↓int	↑/↓		↑↔↓	
Tapering	↑↔	↓↔	↑↔	↓		↑↔	↓↔	↑int & ↓vol	↓/↑	↑	↑↔	
Detraining	↑↔	↓↔	↑	↓		↑↔	↑	↓/no training	↓/↑	↑↔	↓	
**Overreaching/ Overtraining (OR/OT)**												R: (Fry and Kraemer, 1997; Lehmann et al., 1998; Buchheit, 2014); C: (Hottenrott and Hoos, 2017); M: Israel, 1982 *saturation: ↓HRV, ↓HR & ↓HRV/RR ratio
“Sympathetic” OR/ OT	↑↔	↓	↑↔	↓		↓	↑	↑int & ↓↔vol	↑/↓	↓	↓	
“Parasympathetic” OR/ OT	↓	↑↓*	↓	↑		↑	↑	↑vol & ↓↔int	↑/↓	↓	↓	
**Team sport training periods**												O: (Boullosa et al., 2013; Oliveira et al., 2013; Naranjo et al., 2015; Flatt and Esco, 2016; Aoki et al., 2017; Malone et al., 2017); M: Bangsbo and Mohr, 2012; ^u^based on unpublished observations; *changes in key players
Training camps	↔	↑	↓	↑				↑	↑/↓	↓↔	↑	
Off-season			↑					↓			↓	
Pre-season	↓↔	↑↔	↓	↑		↓	↑↔^u^	↑	↑/↓^u^		↑	
Start of the season	↔	↑↔	↓	↑			↓^u^	↓	↓/↑^u^		↑	
1st half of the season	↔	↔↓	↔			↔↓^u^	↔^u^	↔^u^	↔↓/↔↑^u^		↔	
2nd half of the season	↔	↓↔	↔				↔↑^u^	↔^u^	↔↑/↔↓^u^		↔↓	
Playoffs/finals*	↔	↓↔	↔↓^u^				↔↑^u^	↑(↔)^u^	↑↔/↓↔^u^			

*HR, heart rate; HRV, vagal-related HR variability (Ln rMSSD, SD1); HRV/RR ratio, Ln rMSSD to RR-interval ratio; HRex, exercise HR; HRR, HR recovery; HRVpost, post-exercise HRV; HRmax, maximal HR; RPE, rating of perceived exertion; su, supine recording; st, standing recording; RSA, repeated sprint ability; vol, training volume; int, training intensity*.

### Methodological considerations

Using appropriate analysis strategies to interpret individual monitoring data is an essential component of successfully implementing athlete monitoring systems in professional and elite settings (Akenhead and Nassis, 2016). However, there is a considerable research deficit in the area of single-case analysis in sport science and, accordingly, there is a lack of systematic methodological comparisons and recommendations (Buchheit et al., 2014). On the one hand, there is a need for theory-driven and evidence-based methods for data processing and making sense of time series in each measure, while on the other hand, several measures must be combined within a theoretical framework and with multivariate analysis techniques (Kellmann et al., 2018). From a scientific perspective, the ideal overall decision-making process incorporates formalized and validated analysis approaches with high prognostic precision. Furthermore, practitioners need to be able to make quick decisions to modify training and recovery strategies when identified necessary (Starling and Lambert, 2017). Therefore, analysis concepts and methods that enable informative and intuitive visualization are crucial to inform and impact the coaching process (Bourdon et al., 2017; Buchheit, 2017; McGuigan, 2017; Robertson et al., 2017; Thorpe et al., 2017; Heidari et al., 2018). In this regard, the work of Will G. Hopkins on interpreting changes in athlete monitoring (Hopkins, 2004) has had significant impact on current analysis approaches and recommendations in sports research and practice (Akenhead and Nassis, 2016; Buchheit, 2016; McGuigan, 2017; Robertson et al., 2017; Thorpe et al., 2017; Coutts et al., 2018; Kellmann et al., 2018). However, critical evaluation and comparison of the proposed approaches is still pending. In this section, we briefly discuss some of the available analysis concepts, methodological approaches based on univariate data, and possible multivariate strategies to evaluate HR monitoring data.

#### Assessing meaningful change

The overall objective of monitoring training response is to identify meaningful changes to adjust training and recovery prescription, when necessary. To evaluate the importance of an observed change, the measurement accuracy or uncertainty of the observed response, as well as the magnitude of the response, must be considered (Hopkins, 2004; Buchheit, 2014; Thorpe et al., 2017). The *minimal detectable change* refers to changes that are larger than the typical within-subject variation in a measurement, which includes technical error as well as biological variation, and which is usually estimated by measures of reliability (McGuigan, 2017; Thorpe et al., 2017; Hecksteden et al., 2018). However, establishing this threshold requires a normative, and therefore to some degree subjective, determination of “acceptable” error rates (see Hecksteden et al., 2018 for discussion). In this regard, monitoring parameters are commonly rated as *useful* or *sensitive* based on providing high reliability and, therefore, low (random or unavoidable) test-retest variation (i.e., *noise*), which is typically measured as the standard error of measurement (i.e., *typical error*, TE) and often expressed as CV in %. Although a low measurement error is required to identify small observed changes as *true* changes (e.g., changes that are larger than the TE), the magnitude of change that can be expected or elicited by an intervention (i.e., *signal*) is of equal importance. Therefore, it is preferable to judge the sensitivity in a measure by evaluating the signal-to-noise ratio (Buchheit, 2014).

Furthermore, the *smallest worthwhile change* [SWC, also *minimum (clinically) important difference*] describes the minimal change in a measurement that results in a practically meaningful enhancement in sport-specific or competitive performance (Hopkins, 2004) (e.g., a change larger than 1/3 of between-competition CV in individual sports to substantially increase chances of winning a medal, or ~0.03 s for 20-m sprint time in soccer to be ahead of the opponent to win a ball; Buchheit, 2018). Two main concepts may be distinguished when determining the SWC: distributional and anchor-based approaches (Thorpe et al., 2017).

In distributional approaches, monitoring data are evaluated in reference to within-group and/or within-athlete variation, which is commonly done by data-transformation (i.e., Z-Scores) and defining (usually arbitrary) thresholds for trivial vs. substantial variation (e.g., Z-Score >1; Akenhead and Nassis, 2016; McGuigan, 2017). In the former case, an athlete's score or response is compared to the reference group (Julian et al., 2017) and therefore strongly dependent on the group's level and heterogeneity in performance. The latter could be described as a within-athlete distributional approach, typically rating observed values/changes as meaningful when located outside the “normal” fluctuation around the individual mean (Akenhead and Nassis, 2016; McGuigan, 2017). Also, week-to-week changes may be expressed as standardized differences [e.g., week-to-week change divided by weekly standard deviation (SD); (Stanley et al., 2015)].

In contrast to distributional approaches, anchor-based approaches rely on the association between the observed measure and an external (criterion) measure of interest. For instance, a certain amount of (change in) training load, which is associated with increased injury risk (Soligard et al., 2016). Ideally, the assessment of training response incorporates an estimation of an individual confidence interval (or remaining uncertainty) in relation to the SWC (Hopkins, 2004; Hecksteden et al., 2018). For example, practitioners can use an online spreadsheet^1^ to analyze individual changes considering the TE and a (normative) SWC (Hopkins, 2000).

In absence of a sound theory or corresponding empirical observations, changes in resting HR measures are commonly evaluated in reference to the individual within-athlete variation (i.e., SD: standard deviation) in a period of “normal” training, (Buchheit, 2014; Plews, 2014), as they have no direct link to (aerobic) performance (Buchheit, 2017). However, the choice of the threshold value, which in this case is a fraction or a multiple of the SD, is highly arbitrary and subjective, and thus depends on the individual response profile and how conservative the coaching or decision-making should be (Buchheit, 2017). In contrast, the relationship between exercise HR and (aerobic) performance is quite strong, and an empirical SWC of 1% in submaximal HRex was suggested, as it may correspond to a meaningful change in (aerobic) performance (Buchheit, 2014, 2017).

In athlete monitoring, there are also other analysis methods that cannot be clearly assigned to the concepts of *minimal detectable change* or SWC. In training load management, it has become best practice to evaluate short-term (*acute*, usually ~5–10 days) and long-term (*chronic*, usually ~4–6 weeks) accumulated loads using (exponentially weighted) rolling averages and acute-to-chronic ratios (Bourdon et al., 2017). Also, mid- to long-term changes and trends could be evaluated with (linear) trend analysis (i.e., the slope of the regression; Plews et al., 2012; Hopkins, 2017; Sands et al., 2017). Moreover, a more advanced approach was recently introduced by Hecksteden et al. (2017), using Bayesian statistics to compile individualized reference ranges to differentiate between two states of muscle recovery. Group-based reference ranges (i.e., priori distribution) were combined with repeated individual measures to generate individual posterior distributions for each recovery state (Hecksteden et al., 2017; a spreadsheet is provided online by the authors). In summary, although a variety of analysis concepts and methods have been described, there is only a negligible number of studies that systematically compare different analysis approaches (Buchheit et al., 2014; Hecksteden et al., 2018). Moreover, it remains unclear whether and how reference values (e.g., baseline mean and SD or TE) need to be adjusted over time since, among other elements, measurement variability and error are likely training-phase dependent (Taylor et al., 2016). For example, we are only aware of one study that (arbitrarily) updated the individual HR(V) reference values after 4 weeks of training (Vesterinen et al., 2016).

**Figure 3:**
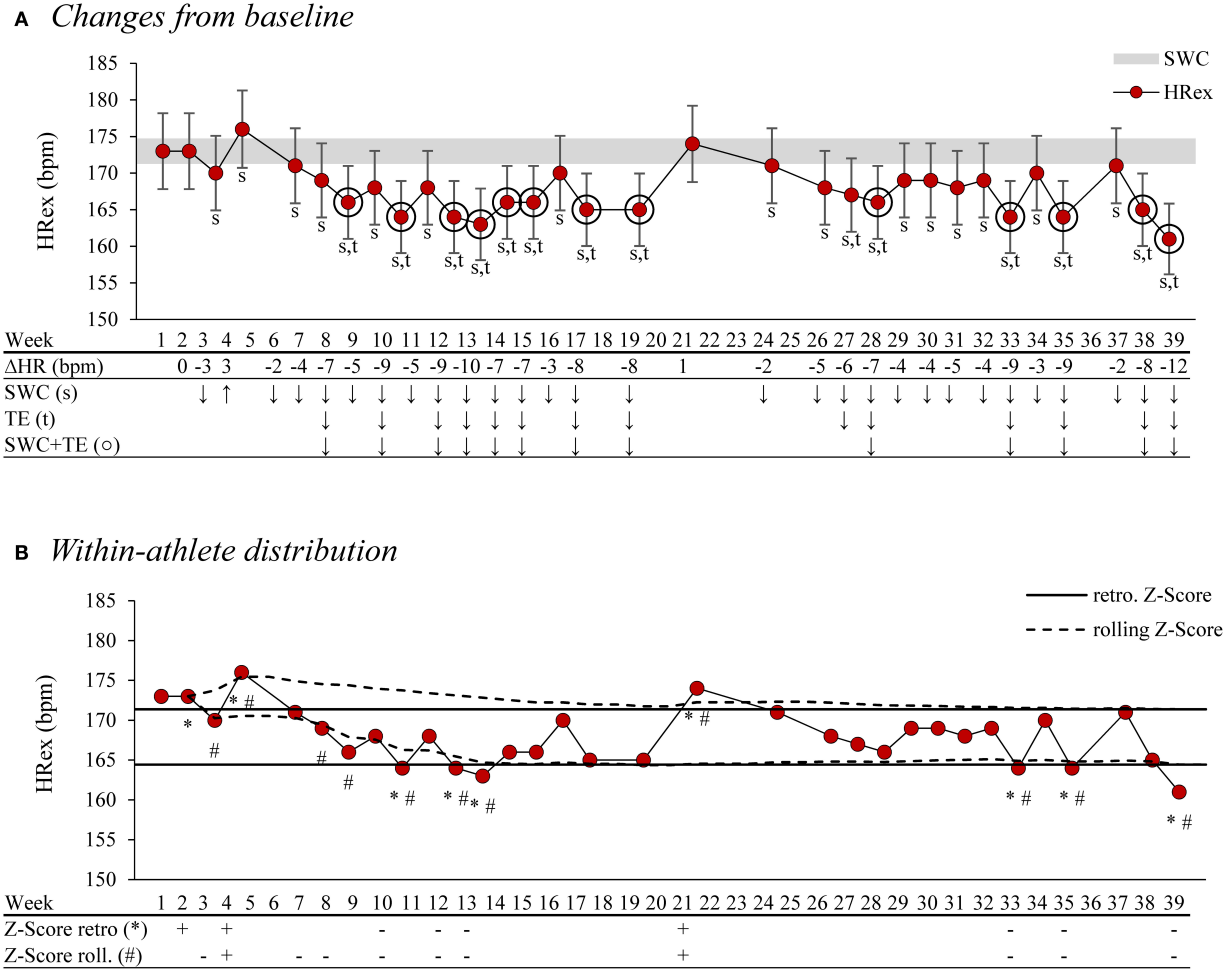
Example of visualization and comparison of different analysis concepts and methods for assessing meaningful change in weekly exercise heart rate (HRex) in a semi-professional basketball player over an entire season. HRex was assessed on a weekly basis using a submaximal shuttle run during the warm-up (see Figure 1). In **(A)**, changes from baseline level (average of first 4 weeks of the preparation period) are rated and highlighted as *meaningful* with three different methods: First, when changes are larger than the smallest worthwhile change (SWC, gray horizontal bar, *s*), second, when changes are larger than the typical error (TE, error bars, *t*), or third, when changes are larger than both (SWC+TE, *circle*). The values for the SWC (>1%) and the TE (>3%) are derived from Buchheit (2014). In **(B)**, changes are analyzed with two within-athlete distributional approaches [Z-Scores: individual mean ± standard deviation (SD)]. The values are rated and highlighted as being *meaningfully* deviated when Z-Scores are >1. In the first approach, Z-Scores are calculated based on the entire data set (solid horizontal lines, *), which represents a retrospective analysis after the data collection was completed. In the second approach, Z-Scores are calculated on a “rolling” and additive basis and with all data available at each point in time (dashed lines, #). This likely represents a more realistic approach in sports practice, as monitoring data are analyzed as soon as available and therefore based on a steadily increasing data set. The analysis concepts and methods visualized illustrate a considerable disagreement between methods and concepts. Symbols: ↓: below baseline, ↑: above baseline, –: 1xSD below the mean, +: 1xSD above the mean.

Figure 3 visualizes different analysis concepts and methods and their effects on rating observed changes as *meaningful*. This example highlights the necessity of a systematic evaluation of the suggested analysis methods and concepts since there is considerable disagreement between approaches (see also Hecksteden et al., 2018 for a detailed discussion).

#### Multivariate approaches

A common *multivariate* approach in HR monitoring is a parallel inspection of several markers in combination with simple decision rules. For example, if RPE during and HRR following submaximal exercise are (clearly) elevated, the athlete is likely fatigued (Lamberts et al., 2011). Typically, either each marker, or a minimum number of markers (e.g., at least 2 out of 3), are required to change beyond predefined cut-off values to be interpreted as substantially deviated (Lamberts, 2009). Rather than analyzing markers in a dichotomous fashion (above- or below-threshold), a continuous combination of different markers as ratios (e.g., HR/RPE, Ln rMSSD/RR) is also often proposed (Buchheit, 2014; Halson, 2014; Bourdon et al., 2017). Moreover, visualizing individual response (pattern) with spider diagrams illustrates another valuable and more insightful alternative to ratios since they display the magnitude of change in every single measure and allow the assessment of changes relative to each other when data are appropriately scaled (Julian et al., 2017).

However, the gradual or hierarchical evaluation of variables in the structure of flow charts (Plews, 2014) or closed-loop models (Kiviniemi et al., 2007; Gabbett et al., 2017) appears somewhat advanced. In this context, the so-called (*fast-and-frugal) heuristics* approach (Raab and Gigerenzer, 2015) provides an attractive opportunity to organize several markers, both structurally and content-wise (i.e., decision trees). At the same time, such heuristics represent an intuitive and simplistic strategy, which reflects fast and practical decision-making in (sports) practice in situations with high uncertainty since only data on a limited number of relevant influencing factors are available (Raab and Gigerenzer, 2015; Jovanovic, 2017). They emerge in the form of (*fast-and-frugal*) decision trees and consist of three main factors: search rules (where to look for information), stopping rules (when to end search) and decision rules (how to make a decision, Raab and Gigerenzer, 2015). However, although “heuristical” interpretation and decision-making appears appealing in general, the application of *fast-and-frugal decision trees* in HR monitoring is still largely limited by the previously discussed research deficits (e.g., inconclusive association between HR measures and training load, fatigue, and fitness or performance; see sections Limitations of Univariate HR Monitoring and Training Context is Key).

Obviously, there are more advanced and complex multivariate analysis methods than the previously mentioned *simple* approaches available. For example, the current training research also suggests the use of multiple (logistic) regressions (Weiss et al., 2017), generalized estimating equations, neural-networks (Pfeiffer and Hohmann, 2012; Bartlett et al., 2017), or modeling techniques based on the original systems-theory model by Banister, developed in 1975 (Perl and Pfeiffer, 2011). Although these advanced concepts are scientifically promising and probably superior to simple or linear concepts, a more detailed discussion is beyond the scope of this report as we are only aware of one investigation using such an advanced multivariate approach to analyze athletes' training response with HR measures (Lacome et al., 2018). Therefore, a broad implementation in sports practice in the near future seems difficult to achieve (Bourdon et al., 2017).

## Practical decision-making with HR monitoring—case examples

This section aims to provide two case studies that illustrate how short- and long-term responses in HR measures could be contextualized and analyzed in a multivariate fashion, using a heuristics approach to guide training and recovery prescription. For this purpose, we first differentiate between the analysis of short- and long-term changes and further define the training context. For simplicity, we distinguish between *training* and *recovery* periods. Training periods are defined as constant or increasing training loads, whereas recovery is characterized by training load reductions or rest. These initial determinations specify how observed changes are interpreted and, therefore, how decisions are made (i.e., *decision rules*). Based on the previously presented research (Table 1), a multivariate analysis of HRex in combination with the rating of received exertion (RPE) might provide adequate information to interpret an athlete's training status (i.e., *search rules* and *stopping rules*) in the following case examples.

**Figure 4:**
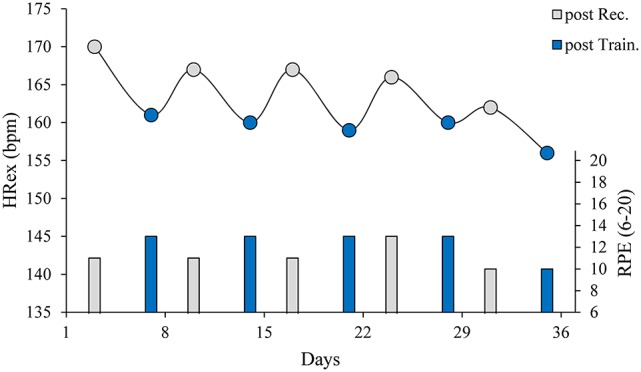
Short-term changes in exercise heart rate (HRex) and rating of perceived exertion (RPE) in an elite, male badminton player (20-year-old) throughout a preparatory period. HRex (circles) and RPE (bars) were assessed on Mondays (post Rec., gray symbols) following 2 days of pronounced recovery, and on Fridays (post Train., blue symbols) following four consecutive days of training (with two sessions on several days) using a submaximal shuttle run (~1, 1, and 3 min at 8.2, 9.6, and 11.0 km/h, respectively; 12.8 m shuttle length) during the warm-up of the morning sessions. HRex was consistently reduced on Fridays (mean ± SD, −7 ± 1 bpm) and increased on Mondays (+5 ± 2 bpm), which may be interpreted as a result of short-term changes in training load between tests. Similarly, RPE during the shuttle runs was typically increased on Fridays and decreased on Mondays. When applying the presented heuristical logic to decision-making, in most cases the obvious conclusions are drawn corresponding to the general training plan: After several consecutive (intensive) training days, the training load should be reduced in the following days to encourage recovery, as the reduced HRex, and the increased RPE indicate acute fatigue. Likewise, the increased HR and reduced RPE on Mondays indicate recovery, which supports a resumption of (intense) training. However, according to the presented logic, one could have deviated from the training plan at two points in time: On day 24, the relatively high RPE indicates an incomplete recovery, and consequently further facilitating of recovery strategies or at least a reduction in planned workload seemed appropriate. In contrast, the low RPE and the somewhat less severe decline in HRex on day 35 point to the possibility of continuing to tolerate high training loads at least for another training session. Furthermore, the overall decline in HRex over the training weeks, while maintaining a constant or slightly decreasing RPE, indicates positive adaptation and appropriate training periodization.

In the first example (Figure 4), an elite, male badminton player was monitored twice per week using a submaximal shuttle run throughout a preparatory period. Although the player is specialized in the *(mixed) Doubles* discipline, badminton is typically classified as a racket sport, not as a team sport. There are, however, great similarities in the training structure and training demands to those in team sports, since different domains, such as endurance, strength, power, speed, and technical and tactical elements are concurrently trained. Accordingly, we are convinced that the observed short-term responses in exercise HR (HRex) and their underlying physiological mechanisms justify transferability to team sport settings. During the training period, we observed a noticeable and consistent pattern in changes in HRex and RPE during a submaximal run in response to the typical weekly training schedules (see Figure 4's text legend for details). In this case, accumulated training loads within the training weeks resulted in reduced HRex and increased RPE, whereas the relief period over the weekend resulted in an increase in HRex and a decrease in RPE. In addition to the short-term fluctuations, an overall decrease in HRex was observed throughout the training period that, taking into account the RPE scores, can be interpreted as a positive adaptation [increased (aerobic) fitness], and thus as an appropriate training periodization. When this observation is transferred to team sports, it highlights the importance of consistent scheduling of testing sessions (e.g., 2 days post game-day), as acute or short-term changes in load can significantly affect HRex response. Furthermore, it may be necessary to consider short-term and long-term changes at the same time when evaluating training programs. Otherwise, in the absence of continuous data, it might be challenging to separate the different types of response (i.e., strain, fatigue, recovery and adaptation) for the interpretation of long-term training responses.

**Figure 5:**
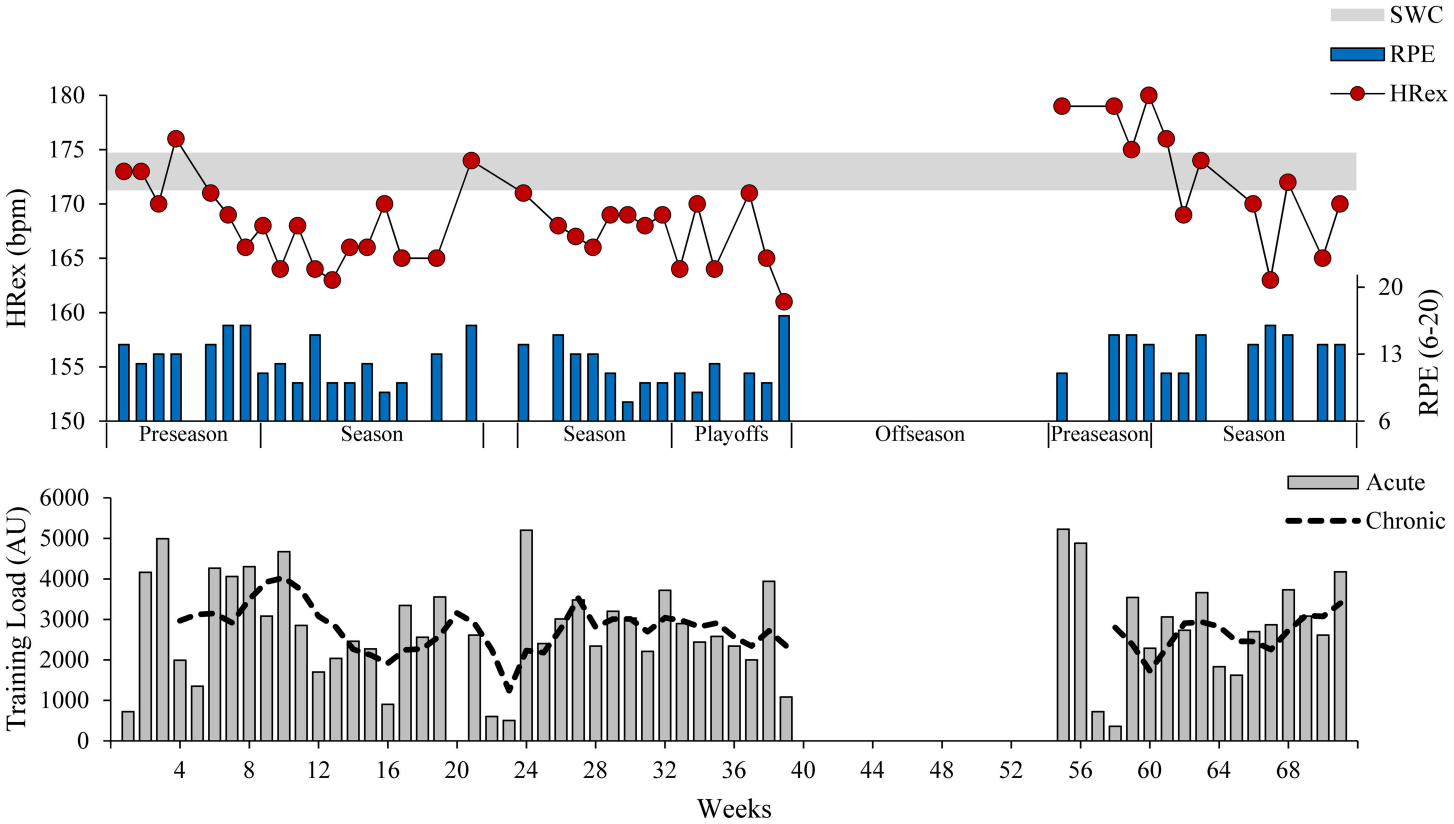
Long-term changes in exercise heart rate (HRex), rating of perceived exertion (RPE) and training load in a semi-professional basketball player (26-year-old, 3rd highest German basketball league) throughout 1.5 competitive seasons. HRex and RPE were assessed on a weekly basis, using a submaximal shuttle run during the warm-up (see Figure 1). Acute and chronic internal training loads were calculated over 1 and 4 weeks of training, respectively (Gabbett, 2016). The gray horizontal bar represents trivial changes from the baseline HRex (average of first four weeks during the first preseason) based on the smallest worthwhile change (SWC; Buchheit, 2014). During the first preseason, HRex displayed a continuously decreasing trend with a concomitantly increasing trend in RPE in response to consecutive weeks of high training load. Since this probably indicates overreaching (Table 1), a (sustained) reduction in training load seems reasonable. As HRex remains substantially reduced during the following months and RPE scores have fallen below the initial values, it can be assumed that the initially reduced load at the beginning of the competitive season allowed sufficient recovery and the training routine at moderate to high training loads can be resumed. In periods of pronounced relief, such as the 2-week winter break (weeks 22–23) and the offseason, there was a significant increase in HR and RPE in both cases. This likely indicates a loss of (aerobic) fitness through detraining, and calls for intensification or resumption of training.

In the second example, a semi-professional basketball player was monitored on a weekly basis using a submaximal shuttle run throughout 1.5 competitive seasons (Figure 5). During the preseason training periods, HRex was markedly reduced both times, likely reflecting positive adaptation. In contrast, in periods of reduced training loads (winter break during weeks 22–23 and off-season), increased HRex in combination with increased RPE indicated (partial) detraining and a loss of (aerobic) fitness. The time course of HRex and RPE response, during the first preparatory period and the beginning of the first season, highlights the importance of training context and multivariate analysis when interpreting long-term changes (see Figure 5 text legend for details). Accordingly, we question some of the conclusions in the HR monitoring literature that show a so-called “counterintuitive” response in overreached athletes (reduced, rather than increased, HRex in fatigued or overreached athletes; Siegl et al., 2017) or “disagreement between studies” (similar changes in HR measures following endurance training periods leading to increased or decreased performance; Bellenger et al., 2016). Using this second example, we suggest that changes in HR measures should be interpreted primarily against the training context, rather than directly projected onto the constructs of fatigue or performance. Therefore, a (sustained) reduction of HRex due to a training period leading to overreaching (likely reduced performance due to fatigue) followed by an adequate relief period should be interpreted as a “typical” training response in the sense of a (positive) adaptation to increased training load. It should not be seen as an “inconsistent” or “conflicting” finding because a performance outcome measured at different times was increased or decreased. This interpretation goes in line with the *fitness-fatigue model*, as a performance outcome is a result of fitness and fatigue effects (Coutts et al., 2018). Accordingly, HRex should be interpreted as a fitness indicator rather than a marker of fatigue or performance.

## Conclusion

As previously suggested (Buchheit, 2014), in team sports, exercise-related measures (HRex, HRR) are probably superior to those under resting conditions (HRrest, HRVrest) as the former have more favorable signal-to-noise and cost-benefit ratios. Moreover, HRex is more reflective of (aerobic) fitness-related training responses than a surrogate marker of performance or fatigue. Therefore, a comprehensive (team sport) athlete monitoring system must incorporate multivariate approaches that further examine training context, fatigue, and sport-specific performance (Kellmann et al., 2018). When athlete monitoring is integrated into a decision-support system, numerous methodological considerations must be addressed throughout the decision-making process. It is necessary to interpret individual training responses by considering the measurement accuracy as well as the *smallest worthwhile change*. As outlined in this technology report, future studies should examine the usefulness of different analytical concepts and methods, as this represents a significant research deficit. Finally, the most appropriate analytical approaches must be implemented in software solutions by wearable manufacturer or software providers to improve the decision-making process in sports practice comprehensively. To provide a starting point, we have developed a conceptual framework to contextualize HR measures, focusing on the time course of training responses as well as training context, and illustrate its application for multivariate interpretation and decision-making using a heuristics approach.

## Ethics statement

The investigations, from which the case studies were selected, were carried out in accordance guidelines of the Declaration of Helsinki. The protocols were approved by the local ethics committees of the Faculty of Sport Science of the Ruhr-University Bochum, Germany or the Ärztekammer des Saarlandes, Saarbrücken, Germany. All subjects gave written informed consent.

## Author contributions

CS prepared the original manuscript, figures and tables. FH, TW, AD, and AF assisted with writing and editing the manuscript, figures and tables. CS, TW, MK, TM, MP, and AF conceived and designed the original observational investigations, from which the case-examples were drafted.

### Conflict of interest statement

The authors declare that the research was conducted in the absence of any commercial or financial relationships that could be construed as a potential conflict of interest.

## Footnotes

^1^sportsci.org/resource/stats/xprecisionsubject.xls (Accessed February 07, 2018).

## Abbreviations

%HRmax: Percentage of maximum heart rate
ANS: Autonomic nervous system
CV: Coefficient of variation
HR: Heart rate
HRex: (Submaximal) exercise heart rate
HRmax: Maximum heart rate
HRR: Heart rate recovery following (submaximal) exercise
HRrest: Resting heart rate
HRV: Heart rate variability
HR(V): Heart rate and heart rate variability
HRVpost: Post-exercise heart rate recovery
HRVrest: Resting heart rate variability
Ln rMSSD: Natural logarithm of the rMSSD
Ln rMSSD/RR: Ln rMSSD to R-R interval ratio
rMSSD: square root of the mean squared differences of successive normal R-R intervals
RPE: Rating of perceived exertion
SD: Standard deviation
SWC: Smallest worthwhile change
TE: Typical error.
